# Clinical application of modified hip joint lateral position in femoral neck fracture

**DOI:** 10.1186/s13018-023-04183-9

**Published:** 2023-09-18

**Authors:** Haitian Liu, Enpeng Gao, Zhiwei Zhong, Wenjuan Wu, Zuzhuo Zhang

**Affiliations:** 1 Department of CT/MR, Hebei Medical University Third Hospital, No. 139 Ziqiang Road, Shijiazhuang, 050051 Hebei Province China; 2 Department of Radiology, Hebei Medical University Third Hospital, No. 139 Ziqiang Road, Shijiazhuang, 050051 Hebei Province China

**Keywords:** Modified lateral view of hip, Cross-table lateral view, Radiography, Femoral neck fracture

## Abstract

**Background:**

To show the femoral neck better in hip lateral view of X-ray, we design a modified hip lateral view and then investigate the value in femoral neck fractures.

**Methods:**

CT images of 10 normal hip joints for 3D reconstruction were selected, the Mimics Medical 21.0 was used, and rotating the proximal femur was to find the most suitable angle for showing the femoral neck well, designed the modified lateral view according to this angle. We collected 35 healthy cases and 35 femoral neck fractures as the normal and fracture group. And two groups were all taken hip anteroposterior view, cross-table lateral view and modified lateral view, which were analyzed by two radiologists to score the anatomical structures of the articular surface, femoral head, head neck junction, femoral neck, basal region and intertrochanteric region. Friedman test was used to analyze the score of femoral neck at different angles. T test and Wilcoxon signed-rank test were to compare inter-groups.

**Results:**

The modified lateral view was designed as follows: The subjects were supine, with the sagittal axis biased toward the healthy side at an angle of approximately 20° to the long axis of the examination table, the hip joint flexed at 45°, the lower extremity abducted at 40°, the centerline inclined 45° toward the head and the centerline aligned with the center of the groin. The modified lateral view showed the femoral head, head neck junction and femoral neck more clearly than the cross-table lateral view, but the cross-table lateral view showed the femoral neck basal and intertrochanteric region better. In addition, the time of taking the modified lateral view was significantly less than the cross-table lateral view (normal group: 0.789 min ± 0.223 vs 0.623 min ± 0.207, P < 0.001; fracture group: 1.131 min ± 0.362 vs 0.946 min ± 0.390, P < 0.001).

**Conclusions:**

The modified lateral view can obtain a standard sagittal image of femoral neck, which can show the dislocation and angulation of the sagittal femoral neck fracture clearly, and improve the accuracy of diagnosis. And it is more convenient and easier for patients to cooperate, which is worthy promoting and applying in clinical work.

## Background

With the development of medical technology, perioperative and postoperative management concepts [1, 2], the treatment methods and surgical plans improved [3, 4], and the prognosis of postoperative patients with hip fractures has improved [5]. However, the huge economic and medical burden it brings to families and society cannot be ignored. As an important component of hip fractures, femoral neck fractures have gradually attracted public attention.

Femoral neck fracture is a common orthopedic traumatic disease. It is mostly associated with a fall, with other risk factors, including decreased bone mineral density, reduced level of activity and chronic medication use. Mostly seen in elderly people. Severe complications, including femoral head necrosis and fracture nonunion, will affect patients’ functional independence and daily living [6–8].

The medial femoral circumflex artery and lateral femoral circumflex artery are mainly distributed on the femoral neck surface of the middle and upper segment, which are the main supplying vessels of femoral head. Femoral neck fractures are prone to damage these vessels, leading to femoral head necrosis and fracture nonunion [9, 10]. Therefore, accurate diagnosis is very important for orthopedic clinicians and patients. X-ray photography has the advantages of simple operation, low radiation dose, mature technology and relatively low cost, which plays an important role in the diagnosis and postoperative evaluation of femoral neck fracture, and is the preferred imaging examination in hip joint disease examination [11–13]. Commonly used photography positions include hip anterior–posterior and cross-table lateral views [14–16]. The cross-table lateral view is complex, especially for patients with hip injuries. X-ray photographs are two-dimensional images with tissues and structures overlapped. Therefore, it is always diagnosing the femoral neck fracture inaccurately, relying on the anteroposterior view (AP) simply [17]. And the study of Chen et al. [18] also found that all of the Garden I femoral neck fractures diagnosed only by hip antero-posterior view were verified to be Garden II fractures under CT examination. In the clinical practice, we found that lateral view as the supplement of AP view could be better for diagnosing femur neck fracture. But conventional cross-table lateral view could not show the femur neck well. Although CT and MRI can be used for diagnosing femoral neck fracture more reliably, they are with high examination cost, inconvenience or larger radiation dose than X-ray photographs [19–21]. Therefore, it is necessary to make a definite diagnosis by X-ray imaging.

In order to reduce the difficulty of lateral hip photography and clearly show the lateral femoral neck, we performed angle analysis using the skeletal 3D reconstruction technique of Mimics Medical 21.0 software to determine the best angle for observing the lateral position of femoral neck, then based on which the modified lateral position of hip was designed. And we evaluated the effect of the modified lateral position and the conventional cross-table view in femoral neck projection.

## Materials and methods

### Postural design

**Fig. 1:**
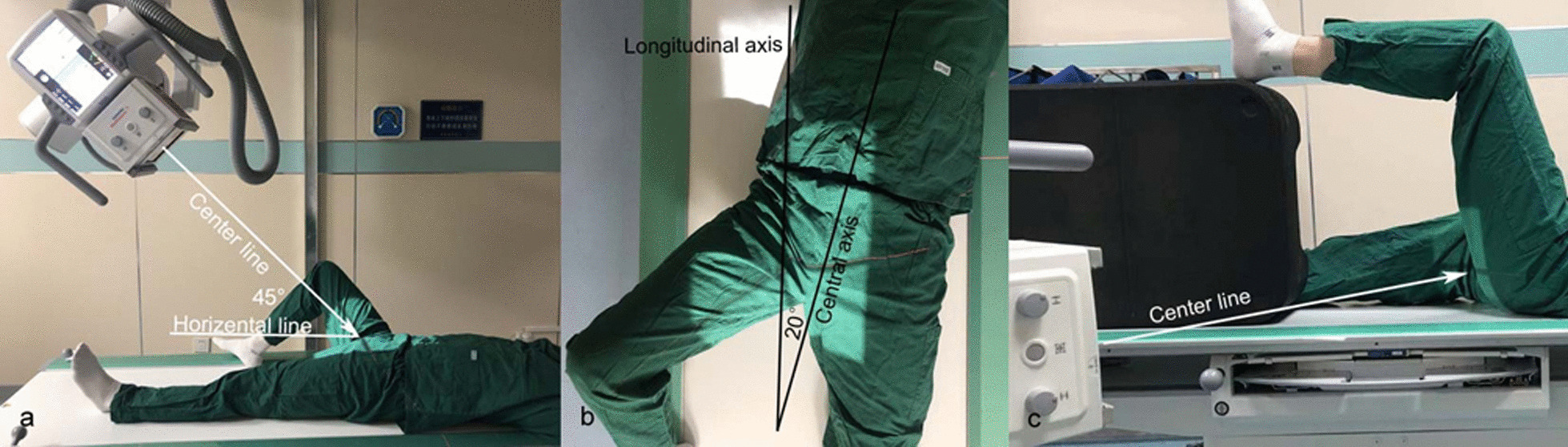
Modified lateral view and cross-table lateral view of hip. **a**, **b** Modified lateral view: the hip flexor 45°, abduction 40°, the body rotation 20° to opposite, and the centerline tilting 45° to the head. **c** Cross-table lateral view: the lower limb of opposite side raises up, and the center line horizontally passes through the hip

CT data of 10 normal hips were collected for the establishment of 3D hip models. Hip scans were performed on all subjects using 128-row spiral CT (Siemens CT SOMATOM Definition Edge) with the following scan parameters: 120 kV, 320 mA, 512 × 512 matrix, layer thickness 1.0 mm. The imaging data are stored in DICOM format. Each set of thin-layer CT data was imported into Mimics 21.0 software, and the 3D model of hip was reconstructed by threshold analysis, region growth, mask repair and other commands. Using the movement command in the software, the hip position was adjusted and the femur was rotated and analyzed. The display of the femoral neck area was simulated in the state of 45° of hip flexion (femur at an angle of 45° to the horizontal plane), and the femur was observed at 25°–60° of abduction, respectively, and scored every 5°. Two imaging experts with senior titles evaluated them and scored according to their display of femoral neck (3-point scale: 3 points for well display, 2 points for partial display and 1 point for poor occlusion) and then screened the angle of femoral abduction when the femoral neck area was best displayed. According to the result a modified lateral view of hip was designed (Fig. 1a, b).

### Image acquisition

In this study, healthy subjects and patients with femoral neck fractures were recruited to perform radiography in different positions. Inclusion criteria for the normal group: ① patients more than 18 years; ② those who were informed and cooperated with the study. Inclusive criteria for the fracture group: ① patients more than 18 years; ② femoral neck fracture diagnosed by AP view or CT; ③ those who knew about the study and cooperated with the study. Exclusion criteria: ① intertrochanteric fracture; ② patients with more underlying diseases and poor systemic condition that cannot be tolerated; ③ multiple fractures, open fractures, pathological fractures, old fractures, etc.; ④ psychiatric disorders and communication disorders; ⑤ those who refused to participate in the study.

All subjects underwent DR examinations of hip joint, including anteroposterior view, cross-table lateral view and modified lateral view. The cross-table lateral view enables the healthy side of the subjects to bend the hip to avoid shielding the affected side of the hip, the affected side of the lower limb rotates 15° inward, and the centerline is aligned with the root of the femur (Fig. 1c). When it is difficult to diagnose the femoral neck fracture through the anteroposterior view, patients need to perform hip CT scanning. Two mid-level technologists recorded the examination time of photography in different positions of each subject and the success rate of the first photography in both positions. The examination time starts when the subject is lying on the table and ready for examination, and ends when the exposure button is pressed. The success rate of first-time photography indicates the number of examinations that met the diagnostic requirements on the first shot/total number of examinations.

### Image evaluation

Two radiologists with senior titles performed diagnosis and evaluation of the images of cross-table lateral view and modified lateral view of two groups in PACS (picture archiving and communication system), including the articular surface, femoral head, head neck junction, femoral neck area, basal area and intertrochanteric area. There are 4 grades of structure unvisible, structure faintly visible, structure visible and structure clearly visible, which are given 1–4 points, respectively. In case of disagreement, the final result shall be determined by the third imaging diagnostic expert with senior title and record the superior rate (number of people with 4 points/total number of people) and display rate (number of people with 3 and 4 points/total number of people) of each part.

### Statistical analysis

SPSS 21.0 statistical software was used to analyze the statistical results, and the values of continuous variables were expressed as mean ± standard deviation. In the scoring of 3d model, Friedman test was used to analyze the score of femoral neck at different angles; P < 0.05 was statistically significant. The examination time of a cross-table lateral view position and modified lateral view of hip joint in the two groups were statistically analyzed by paired t test, P < 0.05 with statistical significance. Wilcoxon signed-rank test was used to statistically analyze the scoring results of a cross-table lateral view posture and modified hip lateral view posture of the two groups, P < 0.05 was statistically significant.

## Result

A total of 70 subjects were recruited, including 35 in the normal group and 35 in the femoral neck fracture group. The average age of the normal group was 49.2 ± 17.0 (18–75 years), including 19 males and 16 females. The average age of the femoral neck fracture group was 57.3 ± 13.7 (18–75 years), including 17 males and 18 females.

### Body position design and centerline tilt angle

**Fig. 2:**
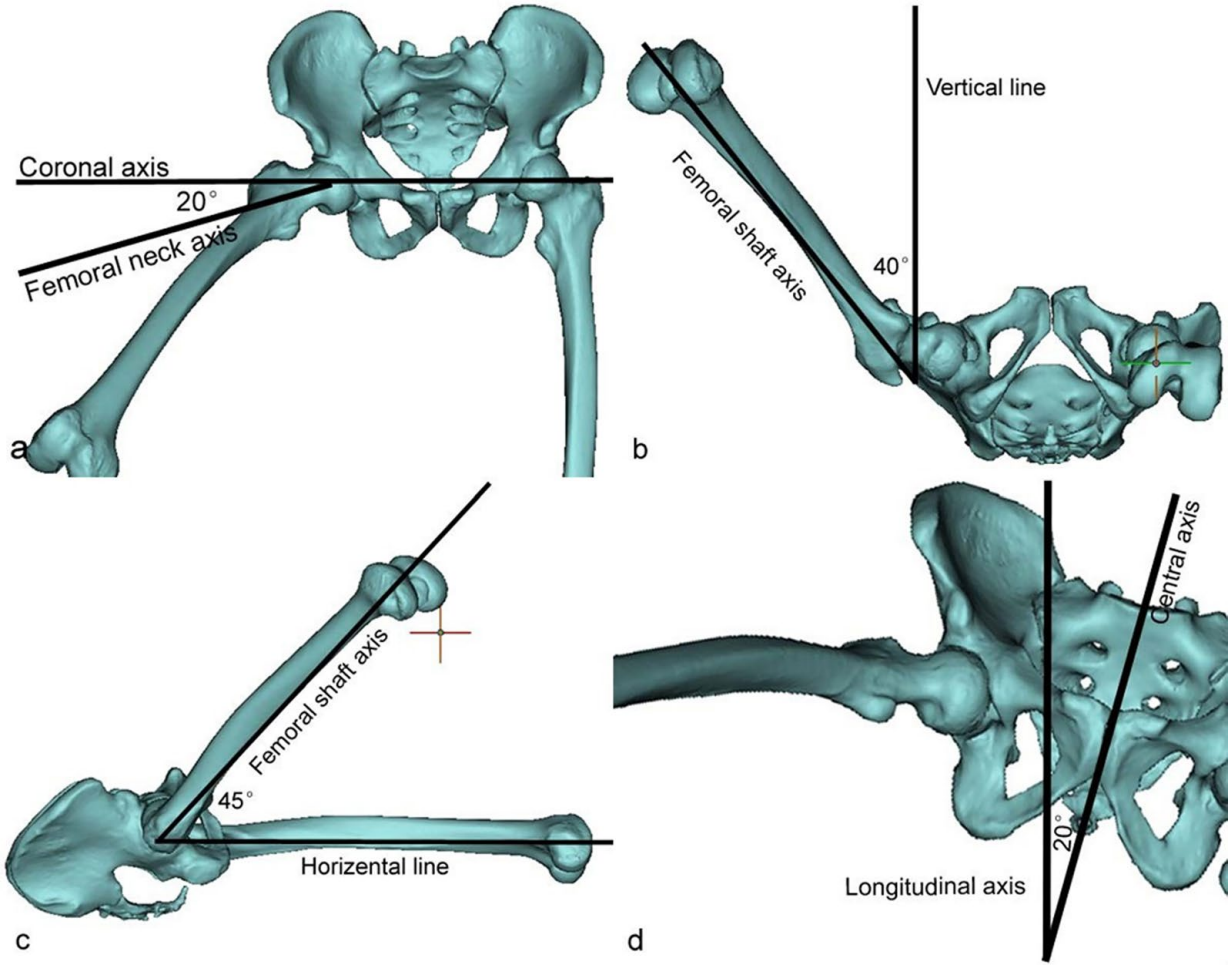
The hip 3D model was reconstructed with Mimics 21.0 software and then rotated the model to get a good view for showing the femoral neck. **a**, **b** and **c** are simulated hip flexion 45°, 40° and 20° in lateral view. **d** Simulating modified lateral view, center line inclined 45°, central axis of body (pelvis) rotates 20° to the opposite side

CT images of 10 healthy cases, 7 males and 3 females (28–53 years, 41.1 ± 8.4), were collected for 3D model reconstruction. When hip abduction 40° in 3D models, the femoral neck was best displayed (χ^2^ = 48.991, P < 0.001, Table 1). According to the results of the 3D model, we designed a modified lateral view: The subject was placed in a supine position, with the body deflected sagittal axis to the healthy side at an angle of about 20° to the long axis of the examination bed, the hip flexed at 45°, the lower extremity abducted at 40°, the centerline tilted 45° to the cephalad side and the centerline aligned to at the center of the groin for incidence (Fig. 2). Siemens Ysio Max DR was used for X-ray image photography. Photographic parameters: tube voltage 80 kV, photographic distance 100 cm, filter grid (+), using ionization chamber automatic exposure technology. Precautions: ① remove all items that may affect the image quality such as casts, dressings, plasters, belts before photography; ② explain the requirements and methods of photography to patients, obtain the patient's cooperation as much as possible and ask him/her to move by himself/herself for those who can move, and for those who cannot move, the operator should move gently, accurately and quickly to reduce the patient's pain.

**Table 1:** Number of Scores of Femoral Neck Display Effect at 5**°** Interval when Femoral Abduction is 25**°–**60**°**

Abduction angle	25°	30°	35°	40°	45°	50°	55°	60°
3 score	0	0	6	10	8	4	1	0
2 score	1	7	4	0	2	5	4	2
1 score	9	3	0	0	0	1	5	8

### Comparison results of practical operability of modified hip lateral view

The shooting time of the modified lateral view group was significantly lower than that of the cross-table lateral view group (normal group: 0.789 min ± 0.223 vs 0.623 min ± 0.207, P < 0.001; fracture group: 1.131 min ± 0.362 vs 0.946 min ± 0.390, P < 0.001), regardless of the normal or femoral neck fracture patients.

Although there was no statistical difference in the first-photograph success rate between the two views in normal groups, the success rate of modified lateral view was higher than that of cross-table lateral view (88.6% vs 94.3%, P = 0.669); similarly, there was no statistical difference in the first-photograph success rate between the fracture groups, but the success rate in the modified hip lateral view group was higher than that in the cross-table lateral view (82.9% vs 91.4%, P = 0.475).

### Scoring results of the cross-table lateral view and modified lateral view

**Fig. 3:**
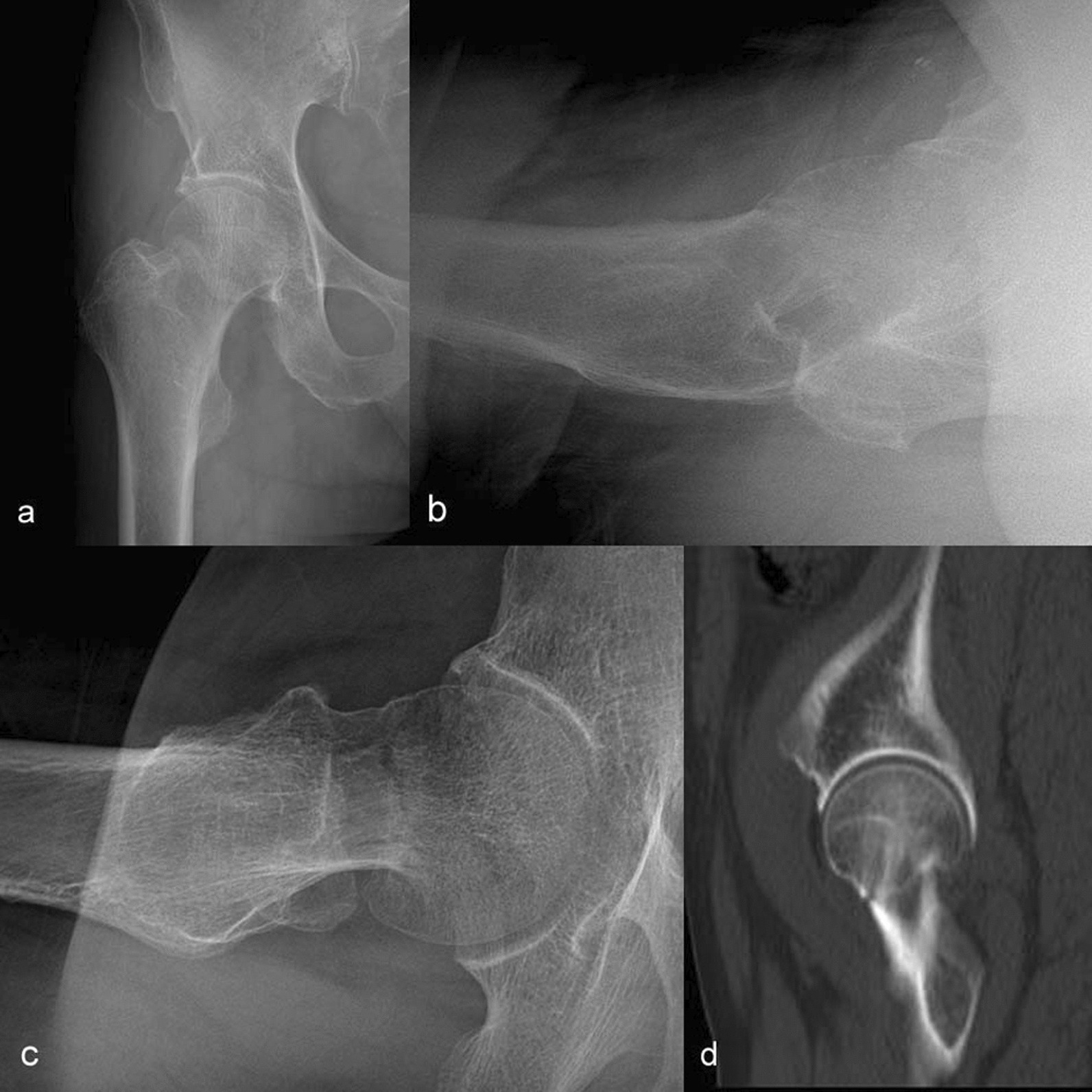
Case 1, a 73-year-old female patient, was admitted to hospital after injury. **a**, **b** show the fracture line of femoral neck faintly anteroposterior projection (AP) and cross-table lateral view of hip. **c** Fracture line of femoral neck clearly in the modified lateral view. **d** CT imaging, it confirms femoral neck fracture

**Fig. 4:**
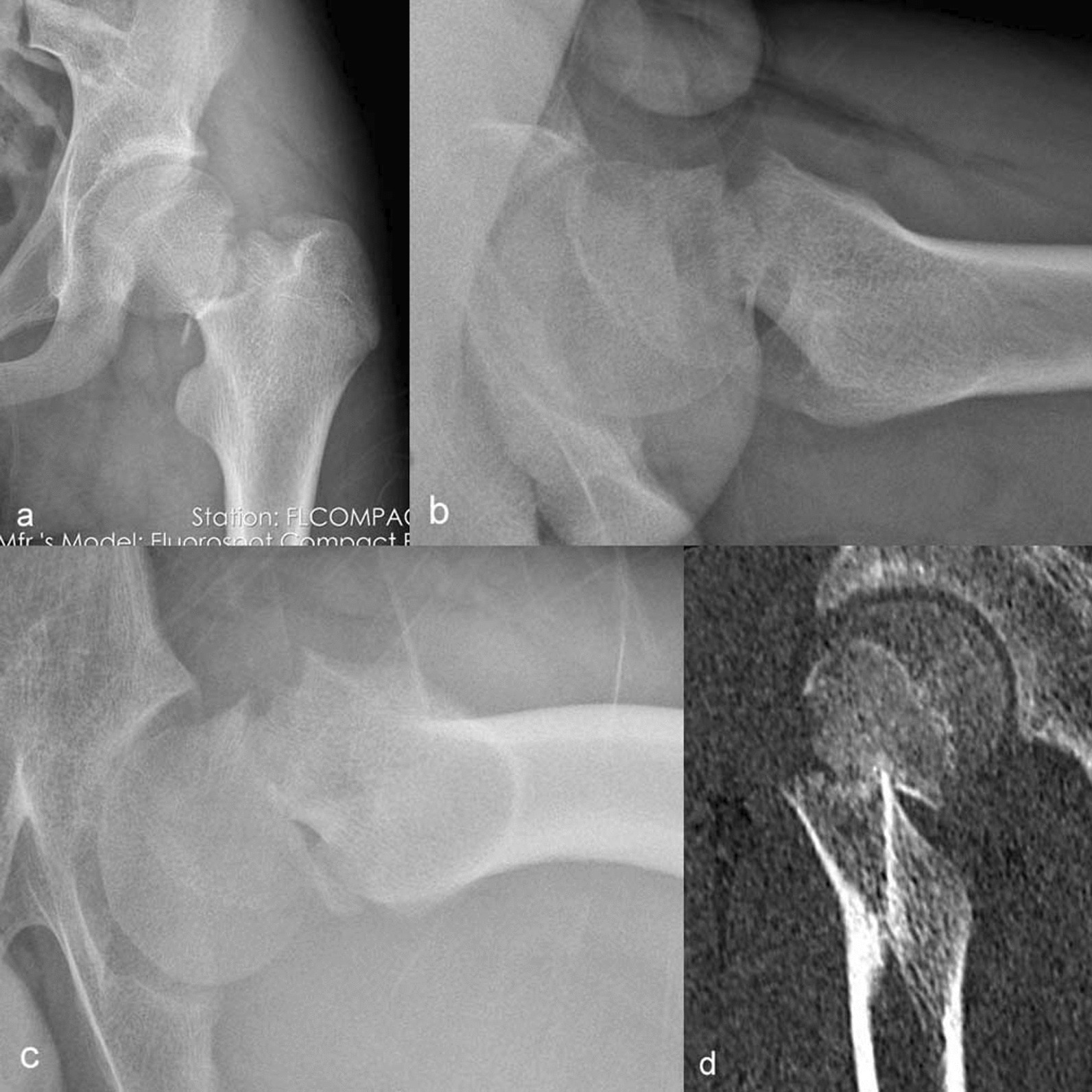
Case 2, a 18-year-old male, admitted to hospital one day after trauma. **a** AP view shows complete fracture of femoral neck. **b** Cross-table lateral view shows angular dislocation at the broken end. **c** Modified lateral view shows more obvious angular dislocation at the broken end than in the cross-table lateral view. **d** CT imaging confirms that the broken end is obviously misaligned and angulated

The results of the normal group showed that the total score of the modified hip lateral group was 663, which was significantly higher than that of the cross-table lateral view, which was 541 (18.94 ± 1.88 vs 15.46 ± 1.93, P < 0.001); the results of the fracture group showed that the total score of the modified lateral hip group was 635, which was significantly higher than that of the cross-table lateral view, which was 534 (18.14 ± 1.87 vs 15.26 ± 2.19, P < 0.001). In addition, in the normal group, the scores and display rates of cross-table lateral view and modified lateral view had significant statistical differences. The display rates of modified lateral view in femoral head, head neck junction and femoral neck were more than 97.1%, significantly higher than the images of cross-table lateral view (P < 0.001, Table 2). In the fracture group (Figs. 3, 4), the display rate of the modified lateral view in the femoral head, head neck junction and femoral neck was more than 91.4%, which was also significantly higher than that in the cross-table lateral view group (P < 0.001, Table 3).

**Table 2:** Display of cross-table and modified lateral views of the normal group in different parts of the hip joint (frequency)

Anatomical site	A Cross-table lateral view						The modified lateral view						P value
	Level 4	Level 3	Level 2	Level 1	Excellent rate (%)	Display rate (%)	Level 4	Level 3	Level 2	Level 1	Excellent rate (%)	Display rate (%)	
Articular surface	2	3	11	19	5.7	14.2	25	8	2	0	71.4	94.2	< 0.001^※^
Femoral head	4	14	15	2	11.4	51.4	27	7	1	0	77.1	97.1	< 0.001^※^
Head neck junction	4	14	13	4	11.4	51.4	28	7	0	0	80	100	< 0.001^※^
Femoral neck region	3	10	22	0	8.5	37.1	31	4	0	0	88.5	100	< 0.001^※^
Basal area	7	20	8	0	20	77.1	3	13	17	2	8.5	45.7	0.021^※^
Inter-rotor area	16	14	5	0	45.7	85.7	0	0	13	22	0	0	< 0.001^※^

^※^Indicates a statistically significant difference between groups

**Table 3:** Display of cross-table and modified lateral views of hip joint in fracture (frequency)

Anatomical site	A Cross-table lateral view						The modified lateral view						P value
	Level 4	Level 3	Level 2	Level 1	Excellent rate (%)	Display rate (%)	Level 4	Level 3	Level 2	Level 1	Excellent rate (%)	Display rate (%)	
Articular surface	3	4	11	17	8.5	20	21	8	5	1	60	82.9	< 0.001^※^
Femoral head	5	8	14	8	14.2	37.1	25	7	2	1	71.4	91.4	< 0.001^※^
Head neck junction	0	11	19	5	0	31.4	28	6	1	0	80	97.1	< 0.001^※^
Femoral neck region	1	12	22	0	2.8	37.1	27	8	0	0	77.1	100	< 0.001^※^
Basal area	9	24	2	0	25.7	94.2	1	9	22	3	2.8	28.5	< 0.001^※^
Inter-rotor area	18	13	4	0	51.4	88.5	0	2	9	24	0	5.7	< 0.001^※^

^※^Indicates a statistically significant difference between groups

However, the display rate of the modified lateral hip view in the normal and fracture groups was significantly lower than that of cross-table lateral view for the femoral neck basal area and intertrochanteric area (P < 0.001, Tables 2, 3).

## Discussion

**Fig. 5:**
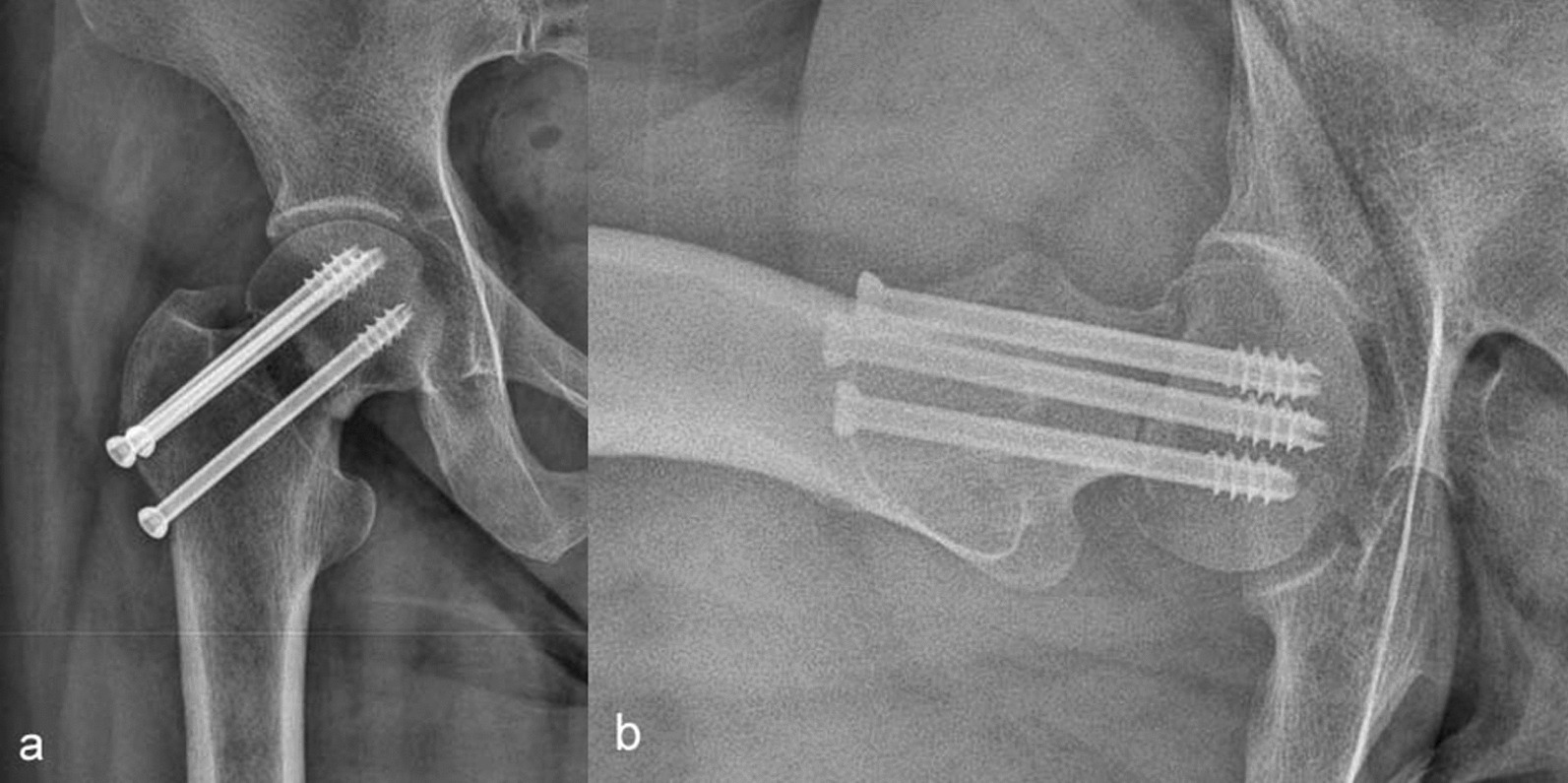
Case 3, a 75-year-old male, one-month reexamination after internal fixation of femoral neck fracture. **a** Anteroposterior projection shows the broken end position and fracture line of femoral neck are not clearly displayed. **b** Modified lateral view shows the position of the fracture end is good

1. Although CT and MRI have higher sensitivity in the diagnosis of femoral neck fracture, traditional X-ray examination is still the first choice with the economy and convenience. Hip anteroposterior and cross-table lateral views are routinely used clinically for hip trauma. As a two-dimensional plane projection, hip anteroposterior provides a lot of diagnostic information in the coronal plane. However, in the sagittal plane, there is no photography position that can show the femoral neck well. In the lateral direction, cross-table lateral view shows the whole proximal femur, but the femoral neck was not fully displayed, especially around the trochanters [18, 22]. Frog position and 45° DUNN views can display the femoral neck well, but the disadvantage is that this position does not display the femoral neck in the direction given by the sagittal plane of the femoral neck, which belongs to the oblique position of the femoral neck [23]. Therefore, a better view for sagittal plane of the femoral neck is necessary and will be of great value for the diagnosis and treatment of femoral neck fractures. In this study, the 3D modeling method of Mimics Medical 21.0 software was used to obtain the appropriate photography position and rotation angle of the modified lateral view by rotating the femur in 3D model. Then, subjects in the normal group and the fracture group were photographed in cross-table lateral view and modified lateral view. Further comparison and verification were made between the two positions. Although the modified lateral view showed poor anatomical structure around femoral trochanters, the display of articular surface, femoral head, head neck junction, especially femoral neck, was significantly better than that of lateral cross-table view in.
2. Although the improved hip position studied by others has some advantages, the iterative reconstruction model of bone is not used to guide the position design. This study simulates the direction of X-ray projection in 3D model to help designing hip lateral view. The 3D model can realistically simulate that in all hip movement, such as bending, extension, adduction, abduction, internal rotation and external rotation, which can control the rotation angle, and it is difficult to achieve by using the actual bone model to simulate the subject's body position. The design of modified lateral view was aimed to make the subjects move as little as possible and be easy to cooperate for the fracture patients. Through clinical observation on patients with femoral neck fracture, most patients with femoral neck fracture were flexed in hip and knee, lying supine or with the affected side on the upper side on a flat bed when examination, so the model was also simulated lying on the back and bending hip by 45° (Fig. 2c), which makes the centerline tilting 45° to the cephalad side to project the sagittal plane of the femoral neck on the horizontal plane; secondly, the femur rotated outward in the plane of the femoral head-greater condyle of trochanter to simulate the lower limb abduction, and then the best display of the femoral neck was obtained at 40° (Fig. 2b); finally, considering the femoral neck will project to the horizontal plane, the 45° X-ray will produce different magnification in the direction of the sagittal axis of the part; therefore, the model was rotated 20° along the sagittal axis to eliminate the 20° angle between the femoral neck axis and the coronary axis (Fig. 2a, d). The design was completed to show the best posture for the femoral neck. This posture is highly acceptable for patients, and a good sagittal images of femoral neck can be obtained.
3. A cross-table lateral view is more complex, requiring the lower limb of the affected side of the subject to be straightened and pronated 15° internally. Most patients with femoral neck fracture report that it is difficult to straighten the affected lower extremity, and pronation will aggravate the pain and make it difficult to cooperate in maintaining the position. If both lower limbs of the trauma patients have fractures, it is almost impossible to complete the examination, and special support is required to fix the mobile plate for detection [24, 25]. The modified posture can be checked with a fixed detector without the cooperation of the other side of the body. The imaging technician only needs to assist the patient to form a rotation angle between the long axis of the body and the long axis of the table and control the tilt angle of the centerline and the incidence position. The patient's hip flexion, knee flexion and abduction can better maintain the posture by themselves, without the technician rushing to operate because he is worried about the patient's inability to adhere. This study shows that the average examination time for improved posture is less than that for cross-table lateral view, and the success rate of the first photography for improved posture is also higher than that for cross-table lateral view. Therefore, the improved lateral position can better cooperate with the fracture patients, reducing the difficulty of cooperation.
4. Penny R et al. [26] calculated the optimal rotation angle of the visualized femoral head neck junction based on CT sectional images and surface data and concluded that internal rotation of the femur at 35° would better show the femoral head neck junction. However, their modification was established in the natural standing position of patients, which is not applicable to patients with femoral neck fracture. The rotation angle of the foot and the torsion of the tibia will affect the angle of the femur. Wan Chin Lee et al. [27] designed a modified axiolateral radiographic hip projection with patients lying flat, the knee flexed, the femur abducted to the bed surface with the bulbous canal tilted 30° to the head side. But it is difficult for patients with femur neck fracture to cooperate, and the display of sagittal view of the femoral neck is not improved. In this study, the modified lateral view was found to be better than the cross-table lateral view in displacement of sagittal view of femoral head and neck. And without the cover of the ischium, femoral head could be displayed clearly in modified lateral position. In the normal group, the display rate of the modified lateral view for the femoral neck can reach 100% and the excellent rate of 88.5% (Table 2), which is much higher than the display rate of the cross-table lateral view. The excellent rate of the fracture group is slightly lower because all the patients in the fracture group are traumatic fracture patients. Different fractures lead to morphological differences from the normal anatomical position, but in the sagittal direction, there are embedded fractures or femoral neck fractures with angled ends (Fig. 4c). The positive view shows that the femoral neck is shorter, resulting from obscuration and unclear observation. The modified lateral position can be clearly displayed. For the inlaid fracture in the coronal direction, the display of fracture morphology in the modified lateral view is not good (Fig. 3c). By taking a modified lateral view, the sagittal fracture of the femoral neck can be observed. Even without CT scanning, it can help to plan the operation plan in advance. And the position could also be used for postoperative examination (Fig. 5), which will be helpful for the assessment of the surgical effect, which will be continuing to research in the follow-up study.
5. This study still has some limitations. ① The design of postural angle was based on a limited sample size of cases retrospectively analyzed, and the abduction angle of the femur derived using bone 3D reconstruction may be biased; ② the modified lateral view of hip requires 45° of hip flexion, and although most patients with femoral neck fracture have reduced pain when maintaining hip flexion, it is still difficult for patients with fracture to cooperate; ③ not suitable for patients with multiple fractures of a unilateral lower extremity. ④ This study also lacks the ability showing the effect of postoperative reexamination of patients effect analysis.

In conclusion, the modified lateral view of hip photography method can obtain the standard sagittal images of the femoral neck, and the joint surface, femoral head, head neck junction, especially the femoral neck, can be clearly displayed, which can clearly show the angular displacement of the sagittal fracture of the femoral neck, greatly improving the accuracy of diagnosis. The modified lateral view is more convenient to operate than the cross-table lateral view and is not limited by the X-ray machine model, and the patients can cooperate more easily, which is worthy of promotion and application in clinical research. The above experimental results can provide effective reference and theoretical basis for clinical photography of patients with femoral neck fracture.

## Data Availability

All the data are available if qualified authors apply for them.
